# Minding the adolescent in family-based inpatient treatment for anorexia nervosa: a qualitative study of former inpatients’ views on treatment collaboration and staff behaviors

**DOI:** 10.1186/s40359-019-0348-2

**Published:** 2019-11-14

**Authors:** Jan-Vegard Nilsen, Trine Wiig Hage, Øyvind Rø, Inger Halvorsen, Hanne Weie Oddli

**Affiliations:** 1 Regional Department for Eating Disorders, Division of Mental Health and Addiction, Oslo University Hospital, Oslo, Norway; 2 Department of Psychology, University of Oslo, Oslo, Norway; 3 Institute of Clinical Medicine, University of Oslo, Oslo, Norway

**Keywords:** Anorexia nervosa, Adolescent, Family-based treatment, Hospitalization, Qualitative research

## Abstract

**Background:**

For some young persons diagnosed with anorexia nervosa, treatment will inevitably involve phases where hospitalization is required. Inspired by the encouraging evidence-base for outpatient family-based treatment for adolescent anorexia nervosa, clinicians and program developers have started to incorporate outpatient family-based treatment principles into higher levels of care. During family-based inpatient treatment, collaborative efforts are largely directed toward the parents of the adolescent. Consequently, the therapeutic focus on the young person is more of an indirect one. With this study we aimed to understand how young persons with lived experience from a family-based inpatient treatment setting, where the adolescents were admitted together with their parents, viewed therapeutic aspects related to staff-patient collaboration and staff-related behaviors.

**Methods:**

Thirty-seven semi-structured interviews of former adolescent inpatients were conducted. Participants’ post-treatment reflections were inductively analyzed by applying a thematic analytic framework.

**Results:**

Based upon user perspectives from a treatment setting highly influenced by a family therapeutic approach, findings revealed that former inpatients prefer tailored treatment and a collaborative approach. Eight subthemes constituting two main themes emerged: 1) *There are no ready-made solutions. Staff should facilitate collaboration by tailoring treatment toward the young person’s perspectives, and 2) Emphasizing skills that matter. Staff should display a non-judgmental stance, educate patients, stimulate motivation, enable activities and prevent iatrogenic effects during the stay.*

**Conclusions:**

This study adds valuable user perspectives to the ongoing work with adapting family-based frameworks into higher levels of care. Clinicians could benefit from viewing their practice from the standpoint of the young person’s post-treatment reflections. From their unique perspectives as having lived experience and hence, “insider knowledge” with a specific treatment situation, clinicians are reminded of the importance of being mindful on the young persons’ views.

## Background

Engaging the young person with anorexia nervosa (AN) in therapy is typically challenged by the disorder’s characteristic ego-syntonic symptom quality and fluctuating motivation for change [1, 2]. As patients often attribute positive values to illness behaviors, it is not surprising that clinicians can find it demanding to uphold a health promoting therapeutic relationship with adolescents with AN [3, 4]. For adolescents with AN, a family therapeutic approach is usually recommended [5]. Even in a well-established evidence-based treatment such as outpatient family-based treatment (FBT) [6], creating and managing fruitful working relationships has been found difficult [7–9].

For some young patients diagnosed with AN, treatment will inevitably involve phases where hospitalization is required. Motivated by the encouraging evidence for outpatient FBT [10], clinicians and program developers have started to incorporate FBT principles into higher levels of care [11–14]. Although these developments could be a promising step for those in need of hospitalization, more research is needed on how to tailor and adapt family-based interventions into inpatient care [15, 16].

Creating and managing a collaborative therapeutic relationship has frequently been positively associated with psychotherapy outcome [17]. This relationship (i.e., the alliance) has been pan-theoretically conceptualized as consisting of three intertwined domains; therapeutic goals, tasks and the affective bond [18]. Within this framework, the quality of the alliance is related to the degree the patient and therapist (i.e., staff) are able to collaborate on therapeutic tasks and goals, as well as the quality of the affective bond [19]. This interpersonal process of co-constructive collaboration is thus embedded in the alliance construct. As a common factor, negotiating the alliance, or collaborating within each of these three domains, lies at the heart of all psychotherapeutic conversations. This relationship has usually been investigated within the therapist–patient dyad and involving adult patients [17, 20]. For adolescents diagnosed with AN, it is both appropriate and necessary to go beyond the therapeutic dyad and involve the whole family in treatment [6, 21]. Hence, in family-based treatments for AN, the emergence of co-existing and multiple working alliances implies further complexity for both creating and managing collaborative relationships.

The parental working alliance is inevitably prioritized during the first phase of FBT. In FBT, parents are charged with the responsibility of managing refeeding and weight restoration. The therapeutic effort converges toward aiding parents to manage this increased responsibility [6]. This more or less all-encompassing emphasis on parents is correspondingly pursued when FBT-principles are adapted to an inpatient setting [12]. Engaging the adolescent in conversations on personal and adolescent-related issues, which may need to be addressed therapeutically, is postponed to the last phase (i.e., toward end of treatment, when weight is restored and the adolescent is able to take back control of eating). Hence, the focus on the adolescent during the initial phases of family-based treatment is toned down.

Although presumably important within a family-based treatment framework, the relationships between aspects associated with the therapeutic alliance and ED outcome are not yet clearly understood [22]. Still, research has shown that the strong parental emphasis embedded in outpatient FBT is mirrored in alliance evaluations, as it is usual to observe higher scores of parental alliance, when compared with the young persons’ scores [23]. There is also some preliminary evidence suggesting that the therapeutic alliance is differentially associated with outcome for parents and the young person [23]. Parental alliance has been associated with weight restoration and treatment retention [24–26], whereas the young persons’ alliance has been associated with psychological measures [23, 24].

Qualitative research on patients’ treatment experiences can both aid treatment development and aid clinicians to tailor interventions [27, 28]. Qualitative research has shown that patients with AN typically prefer treatment to be a joint and collaborative effort and favor therapists who are supportive, non-judgmental, active (i.e., taking initiative), respectful and caring [29–32]. Overall, qualitative research on patient preferences seem to converge toward patients favoring therapists that are skilled in ED management, and able to utilize a wide range of behaviors (i.e., displaying both acknowledged therapeutic stances and capable of multiple ways of intervening), when engaging patients in therapy [30, 32]. Reassuringly, young patients taking part in outpatient family-based treatment seem to appreciate the increased parental responsibility, externalization of the ED and that treatment enables lower degrees of within-family criticism. Still, this research has also shown that in hindsight, adolescents prefer greater involvement in family-based treatment, as important issues are perceived as being neglected [33]. Although quantitative studies of the relationship between therapeutic alliance and ED outcome show mixed results [22, 34], findings suggest that the quality of the therapeutic relationship can be of extra importance for younger patients. In fact, various aspects of the therapeutic alliance have shown stronger relations to outcome for younger versus older patients [22].

The present study was conducted within a family-based treatment context where adolescents are admitted together with parents, and, if appropriate, siblings. Our study aligns with previous qualitative research which has called for additional research to address the perspectives and viewpoints of young AN patients involved in family-based treatment [28, 33]. Our overarching aim was to investigate post-treatment reflections following discharge from a treatment program which, corresponding to family-based treatment, emphasized parents. Specifically, the research questions were a) how do the participants with lived experience from a family-based inpatient treatment experience collaboration with staff, and b) which staff behavior and skills are valued and/or considered important. By prioritizing the young person’s “insider knowledge” with a family-based inpatient program, we aimed to inform ongoing discussions on how to optimize the inpatient setting for those in need of family-based treatment for AN at higher levels of care.

## Methods

This is a qualitative study that forms part of a larger research project with a naturalistic design aimed at investigating the outcome of inpatient family-based treatment within a tertiary ED inpatient unit for adolescents [11].

### Participants

Thirty-seven (64%) of 58 invited former inpatients (33 females/4 males), provided written consent to participate in this sub-study. For the sole participant under the age of 16 (i.e., age of consent) at follow up, parental consent was also provided. There were no significant differences on clinical and demographic variables when comparing participants with non-participants [11]. All had a primary diagnosis of AN, and were admitted together with family members between 2008 and 2014. Prior to the family-based admission, all participants had received outpatient treatment at their local child and adolescent clinic. Approximately three-quarters previously had at least one inpatient admission to their local hospital. Duration of ED prior to the family admission (FA) was on average 2.7 years (range; 0.5–6.0, *SD* = 1.8). Mean age at admission was 15.8 years (range; 12.4–19.5, *SD =* 1.8). The majority (33/37) were admitted voluntarily. Mean length of stay was 20.8 weeks (range; 3–58, *SD =* 13.5), including planned leaves from the ward as part of the treatment program. All families agreed to stay at the hospital with their child during the hospitalization. The mean number of years after discharge to the follow-up interview was 4.5 years (range; 1.3–7.0, *SD =* 1.7). The mean age at follow up was 20.2 years (range; 15.8–25.3, *SD =* 2.6). Thirty-eight percent had received additional inpatient treatment during the follow-up period. At follow up, the majority (65%) had achieved normal body weight, as defined by attaining a BMI ≥18.5 [11]. The mean body weight improved during admissions (7.6 ± 4.3 kg), and the mean BMI-percentile at discharge (21.4 ± 17.8) was in the normal range (i.e., > 12, which corresponds to approximately BMI 18.5 in adults).

Twenty two (59%) participants did not meet the criteria for any DSM-V ED-diagnosis, 8 met criteria for AN, 2 for BN and 5 for OSFED. ED diagnoses at follow-up were determined by using the diagnostic items of the Eating Disorder Examination 16.0 [11, 35].

### Treatment setting

Throughout family-based inpatient treatment, staff actively promotes collaboration with parents. Consequently, the therapeutic focus on the young patient is more of an indirect one. Without adhering to manualized FBT, the guiding treatment principles were inspired by outpatient FBT [6, 11]. The overall treatment focus for the majority of participants corresponds to the first phase in outpatient FBT. The main treatment program features included giving parents increased responsibility for managing meals and weight restoration, externalizing the ED and adhering to a non-blaming and non-etiological stance. The main programming consisted of family therapy, supplementary individual therapy and milieu therapy with the overarching aim of supporting parents to support their child during the stay.

Up to five families were hospitalized at the same time. Although all members of staff assisted families, each patient and family were allocated a multidisciplinary team during the duration of stay. The nucleus of this team consisted of a child- and adolescent psychiatrist working closely with a clinical psychologist, and two or three nurses. The team and family members could consult a clinical nutritionist as needed. Families were typically offered family therapy sessions at least twice a week. Some patients were offered supportive individual therapy in addition to family therapy, and this was arranged in collaboration with the adolescent and parents. Nursing staff had daily scheduled conversations with both parents and the young person, for preparing meals and evaluating the ongoing process, together with spontaneous ad hoc sessions as needed during the day. Patients and parents took part in the weekly treatment meetings. At discharge, all patients and families were referred back to their local clinic for further outpatient treatment.

### Recruitment and data collection

Ethics approval for this study was obtained from the Regional Committee for Medical Research ethics, South East Norway [REK2014/2223]. Thirty-seven participants took part in a semi-structured interview which was administered by a senior researcher, two clinical psychologists, one psychiatrist or a psychiatric nurse. Four of the interviewers had been directly involved in the provision of treatment. Twenty-six of the interviews were conducted on-site at the hospital, seven at the participant’s home, three by telephone, and one in-person elsewhere. All interviews (including telephone interviews) were audiotaped and transcribed verbatim. The qualitative interviews lasted between 30 and 100 min.

### Interview guide

The semi-structured interview guide was originally developed to investigate participants’ post-treatment reflections on a range of issues, and not specifically designed for the sole purpose of this study’s research questions. The interview was structured into three sections: pre-admission, during admission, and post-admission experiences. Main questions used for the present study included, “Looking back, how was the admission for you?” “How did you experience the support from the staff?” “Do you have any ideas on wanting anything to be different during the family-based admission?” and “What should treatment providers emphasize in their work with adolescents with an eating disorder?”

### Qualitative data analysis

All 37 participants were included in the qualitative thematic analysis to allow as much diversity in views as possible. Transcripts were analyzed according to six phases outlined by Braun and Clark [36]. The analysis was mainly informed by an inductive and semantic approach. Inductively analyzing the transcripts meant that we aimed at staying sufficiently long with the raw material to “truly” grasp the meaning of the accounts. Applying a semantic approach implied that the explicit and surface meanings were primarily considered, rather than inferring beyond the content conveyed in the accounts, as would be the case with a more interpretative, implicit approach [36].

First, the first author read all the transcripts several times. To increase familiarity with the material, three of the co-authors read randomly selected interviews. The first author was responsible for coding, identifying and developing the main themes and adjacent subthemes. The analysis was conducted in close collaboration with two of the co-authors (HWO and TWH). Following multiple team discussions, the theme structure was reviewed and discussed, and during the process there were several modifications to achieve a final consensus between all collaborators (i.e., JVN, TWH & HWO) on how the specific labels and structure could best reflect the raw material. Before completion, the first author re-read all transcripts to ensure that the themes captured the material in a reasonable way. The QSR International’s Nvivo11 Software was used in the initial phase of coding [37].

## Results

The thematic analysis yielded 2 main themes and 8 adjacent subthemes (see Table 1) as presented below. Subthemes are illustrated by quotes. The source of each quote is indicated by the participant’s research ID number. Quotes are directly translated from Norwegian to English with only minor revisions to enhance readability.

**Table 1:** Minding the adolescent in family-based inpatient treatment

Main theme 1: There are no ready-made solutions. Staff should facilitate collaboration by tailoring treatment toward the young person’s perspectives	Subtheme 1: It’s not always best to go by the book (*N* = 25) Subtheme 2: Managing the balance between the symptoms and the person (*N* = 18) Subtheme 3: Managing the balance between flexibility and firmness (*N* = 25)
Main theme 2: Emphasizing *s*kills that matter. Staff should display a non-judgmental stance, educate patients, stimulate motivation, enable activities and prevent iatrogenic effects during the stay	Subtheme 1: Beware of stereotypes and prejudice: cultivating respect and curiosity (*N* = 24) Subtheme 2: Exploring and working with personal goals: strengthening the young person’s own motivation for recovery (*N* = 20) Subtheme 3: Providing information and transferring knowledge in meaningful ways (*N* = 15) Subtheme 4: Enabling a shift of focus by providing activities (*N* = 14) Subtheme 5: Addressing and working with covert ED-behaviors at the ward: be attentive and preventive (*N* = 13)

Numbers in parenthesis (N) equals the number of participants’ sharing accounts within each subtheme

### Main theme 1: there are no ready-made solutions. Staff should facilitate collaboration by tailoring treatment toward the young person’s perspectives

The majority of the participants emphasized that treatment must be a collaborative and reciprocal endeavor. Several suggested that treatment teams should aim for developing a novel or unique treatment for each patient and “not do the same thing over again.” Quite a few participants reflected that a more adolescent-oriented approach was needed, and that health care professionals should be mindful of individual differences in needs and vulnerabilities, with flexibility in potential solutions. Many emphasized that treatment teams should integrate the views of the young person into decisions, allowing for a more shared and dynamic decision-making process. The subthemes portray the aspects of collaboration which were valued as especially important.

### Subtheme 1: It’s not always best to go by the book

Participants stressed that treatment should be tailored to fit the individual, family, and their unique situation. Some called for more comprehensive assessment of their specific needs and vulnerabilities prior to the start of treatment. Several reflected that they felt the treatment approach or dominant structures were too predetermined:

*P: … individuality … ehm … yes … be aware that they are different patients … different disorders … and different illness histories … maybe not just do the same thing over and over again … that it is not always … it’s not always best to go by the book … [P60].*

… and others, that treatment has to be wisely adapted, since treatment is not “one-size” fits all:

*P: There is no one way of having an eating disorder. There are as many eating disorders as there are persons suffering from them, so you can never have a book for how you manage “Eve 14 and her anorexia” … there is no … it’s not like that … [P15].*

### Subtheme 2: managing the balance between the symptoms and the person

Several of the participants reflected on the importance of not losing sight of the person behind the symptomatic behaviors. Several emphasized the importance of striking a balance between focusing on the person versus the ED, and echoed the potential negative consequences of an unbalanced approach (i.e., too symptom oriented). Even though the vast majority acknowledged the necessity of weight restoration and managing somatic complications during treatment, many had views similar to P56:

*P: I wish that, at least in certain phases of treatment … that there could have been more focus on me, who I was, and not just how the ED influenced me. I was in pretty bad shape when I was admitted and it became easy, in a way, to not see me … one only saw what was driving me. That was also a frustration I had back then, because I was really suffering and the ED became, in a way, my survival technique and that they in a way just took that from me, without giving me the chance to get better. That was very painful … and … that … yes … I did gain weight during that admission, but I didn’t feel that I had really improved, thinking differently, when I was discharged … [P56].*

Others shared views in line with P10:

*P: I often felt like a number, from week to week … that in a way … it was the number on the scale that decided how it went that week … and that this didn’t relate to how I felt … and when you, or the staff, was most happy … because I had gained … that was the most difficult part for me … [P10].*

### Subtheme 3: managing the balance between flexibility and firmness

Several participants shared their perspectives on rules and routines encountered in the highly structured inpatient setting. Taken together, this subtheme conveys a need to manage the inpatient structure in a more collaborative way to match the perceived needs and vulnerabilities of the individual. Many of the participants preferred that rules be negotiable to a certain extent. Quite a few reflected on the difficulties of adhering to strict rules that did not seem to fit their perceived needs at the time. For instance, being required to participate in mandatory group resting time after meals could be viewed as unnecessary for those without problems sitting still or purging, and possibly promote disengagement or resistance to treatment. However, some participants favored rules, as rules were viewed as necessary and therefore valued:

*P: That I wasn’t allowed to negotiate then … That it was … That I couldn’t do. That was a good thing, because then I gave up on that, and … even if it sounds a bit silly; that you should eat every last bit of that yoghurt … it was … making me safe … […*] *… Ehm … that it was … ehm, that it was … ehm … strict … that was at least making me secure … [P34].*

Whereas others advocated for a more flexible and individualized approach:

*P: I think the rules should be more individually adjusted, so if you don’t have a certain problem, you don’t need to face the same rules as those who in fact struggle with it … [P51].*

### Main theme 2: emphasizing skills that matter. Staff should display a non-judgmental stance, educate patients, stimulate motivation, enable activities and prevent iatrogenic effects during the stay

The second main theme captured 1) the acknowledgement by participants that AN treatment is a highly complex and difficult endeavor, and that 2) staff needs multiple skills within different domains to engage the young person in treatment. While the first main theme captured the participants’ call for modifications and individual tailoring of treatment, the second theme pertained to preferred staff characteristics and skills.

### Subtheme 1: beware of stereotypes and prejudice: cultivating respect and curiosity

Participants emphasized the importance of friendliness and kindness. Some emphasized that years of medical education and extensive clinical experience did not matter if staff did not treat the young person with respect and curiosity. Some remarked that they easily noticed whether staff members were emotionally invested in their jobs, and preferred staff that were highly invested in their work and “not just doing their job to get their salary.” Respect, genuine curiosity, and a non-judgmental stance were all highlighted as important professional characteristics. Some emphasized that they were usually treated with respect and curiosity during their admission, which had boosted treatment involvement.

*P: They were considerate, respected me for who I was. They were attentive, that was of importance too, and I felt in different ways that they understood me, and that I … like, opened up and in ways observed, noticed their reactions. And then I felt even more secure … and, that I could open up even more and more. That I remember as a good thing … [P23].*

Others, however, reflected upon having the opposite experience, being perceived as “yet another anorexic” and stereotyped in generalisms. Quite a few participants cautioned staff against being too “know-it-all”. Participants underscored the importance of staff displaying a genuine interest in getting to know them as people, and understanding the influence the illness had upon their lives, without too much preconception.

*P: … they said things that maybe … as if they knew … said things in ways that sounded like they in a way knew things better than me … and that … They couldn’t know how I felt and how things were for me … And some were maybe generalizing a bit, on how the ED was … because that is individual, for everybody … [P56].*

### Subtheme 2: exploring and working with personal goals: strengthening the young person’s own motivation for recovery

Working with the young person’s own motivation for change was emphasized. Participants acknowledged this was a demanding undertaking, as many recalled being highly indecisive and some even resisting treatment during the admission. However, several participants viewed personal readiness and commitment to change as the most important aspect of recovery, thereby deserving greater attention during treatment. Many participants shared views such as “you have to want to change yourself, to make change happen” or “it was when I decided to change myself that change really started to happen”. Collaboratively exploring and setting personal future-oriented goals were emphasized as important mechanisms to enhance treatment engagement and provide meaningful goals. In hindsight, several acknowledged that identifying personal reasons to recover was a crucial component in the recovery process:

*P: … that [motivation] is the most crucial aspect, right? in the treatment of eating disorders … so … that is the most important … when motivation emerges you have to do anything to maintain it … because it is so crucial and rare … that is what makes eating disorders so difficult to treat … that it is the only disorder you don’t want to get free from … that’s why motivation is so important when talking about treatment … [P60].*

### Subtheme 3: providing information and transferring knowledge in meaningful ways

Participants emphasized that staff should be highly skilled in providing information and transferring knowledge, for example, on the various somatic and psychological aspects of starvation, purging and excessive exercise. Reflecting back, however, participants acknowledged this might be difficult to accomplish immediately upon admission, as the young person may have little interest, or regard this information as irrelevant during early phases of treatment:

*P: It would have been useful with more information on the physical consequences by being underweight over time, and on how physical and mental states influence each other. Because that is really something I’ve had to discover myself. I don’t think I really got any information … [P10].*

Others reflected on the necessity of advice or information being delivered in a constructive and collaborative manner, not just stated repeatedly as factual information to be trusted:

*P: … You have to make them think … not just tell them to … for example; “you have to eat so and so much” … it wouldn’t be of any help … maybe there and then … but in the end you have to work on the mental part … make them to work on the psychological issues first … that was at least what I did … and after a while the other things will find its way … it is important to find the drive … to answer the questions of “why … should I do this, why should I eat more … why should I gain weight” … and [help them] transcend the fear we all have, of getting fat … and all that … [P22].*

### Subtheme 4: enabling a shift of focus by providing activities

Several highlighted the importance of initiating a variety of activities to engage young patients and shift the focus away from a potentially highly monotonous treatment environment. Shifting focus by providing extracurricular activities also accommodated other important aspects of their daily lives. Some encouraged staff to feel “freer” when engaging the young patient, and not be too afraid to assume the parents’ roles and responsibilities. Rather than requesting activities for the family to do together during the admission, participants appreciated staff-led initiatives, as the feeling of boredom during treatment can represent a vulnerable situation.

*P: Once we went to an amusement park … and we got to live more as normal human beings … [P33].*

Several called for activities beyond the ED-focused treatment schedule, and emphasized the importance of variety:

*P: It was very quiet here. It was helpful when I could go out and go for a walk and things like that … It easily becomes boring when you’re admitted … so I think … It was a small activity room here … but things were very little organized around that … […*] *so maybe a bit more drive from the staff too … to ask whether we should do things … [P31].*

### Subtheme 5: addressing and working with covert ED-behaviors at the ward: be attentive and preventive

Some emphasized that illness behaviors were both maintained and exacerbated within the context of the treatment unit, even during family-based admissions. Examples of illness behaviors included self-induced vomiting, excessive exercise, water loading pre-weighing, and attaching objects to the body to increase weight. Some participants felt that these behaviors were poorly addressed during treatment, and some reported learning new ED-behaviors while hospitalized. Reflecting back, participants emphasized that staff must be knowledgeable about the manifestations of the illness, in addition to potential ways to conceal illness behaviors during hospitalization. Some warned staff to not be too naïve or inattentive to the evident self-destructive forces that can drive a young person with AN during hospitalization:

*P: If I hadn’t had the shirt on, then I couldn’t have put the weight belt on, and maybe they would have discovered that my bladder was completely full … ehm … I think at most I drank 4 l of water … [P32].*

Participants underscored that staff should be aware of, thereby potentially preventing, various illness maintaining behaviors such as water loading, attaching weights, purging, and excessive exercise to burn calories at night or in a private room:

*P: Look more after patients when they are at the loo … mhm … and don’t allow too much solitary time in their room. I was running around continually, to burn calories. It was very exhausting, yet I felt I just had to … [P63].*

## Discussion

This qualitative study investigated the viewpoints of former adolescent inpatients admitted to a family-based inpatient treatment program. Knowledge of how young patients with AN generally experience and perceive various aspects of treatment and staff-related behavior is scarce [28]. Knowledge is especially lacking regarding young patients’ experiences within a family-based treatment approach for AN at higher levels of care [33]*.*

The participants’ reflections revealed that involvement and collaboration are highly valued, along with efforts to individually tailor treatment. They also recognized that staff requires diverse skills to facilitate engagement in treatment. With some exceptions, few viewed treatment as a reciprocal and collaborative experience. Improved collaboration was desired to achieve better balance between the ED versus the person, and to provide sufficient flexibility when negotiating the rules and structures, thereby individually tailoring treatment. Reflecting back on staff-related behaviors, the participants emphasized the importance of showing genuine interest in the young person, rather than an enhanced focus on family processes. Other desired staff-related skills and characteristics included having a non-judgmental stance, educating patients, enhancing motivation, providing activities and preventing iatrogenic effects during the stay.

Findings pertaining to the importance of facilitating a good therapeutic collaboration align with psychotherapy literature documenting the co-constructive nature of therapeutic processes and the importance of negotiating the therapeutic alliance in therapeutic encounters [20, 38]. However, quantitative research investigating the intricate bidirectional relationship between measures of the therapeutic alliance and treatment outcome in ED treatment has shown varied results. Alliance research has suggested that early symptom improvement fosters a positive influence on the alliance in ED treatment, and that the therapeutic relationship can be of extra importance for younger patients, as studies show stronger relations between alliance and outcome for younger versus older patients [22]. Our findings extend prior qualitative research which has shown that patients with EDs often value aspects associated with the therapeutic alliance, preferring treatment as a joint and collaborative effort, as demonstrated in main theme 1 [29–32].

Taken together, our findings shed light on managing complexities, and might suggest the need for a greater degree of tailoring and differentiation when providing family-based inpatient treatment, as there is no treatment program that fits all. Our findings suggest we critically examine whether the inpatient context, with common rules and structures, offers sufficient tailoring to the individual family and young person, an intended hallmark with outpatient family-based therapy [6, 21]. Managing the balance between set structures and sufficient flexibility during hospital admissions is a complex endeavor [28, 39, 40].

The emerging literature on feedback-informed treatment may prove an inspirational source to encourage feedback from young persons during treatment. Ideally, inviting feedback could improve aspects of the working alliance and thus, enhance the feeling of working together during treatment [41, 42]. Still, this is an intricate balance, as we can imagine that invitations to negotiate “the non-negotiables” (i.e., negotiate fixed rules and structures associated with inpatient treatment) may be problematic and in the worst case, fuel the ED (i.e., allowing too much negotiation could prove to be a pitfall). Nevertheless, reconsidering the “non-negotiables” might be more of a question of how, rather than if, we should negotiate with younger persons during family-based admissions to achieve better collaboration.

The second main theme implied that health care professionals and multidisciplinary teams should cultivate diverse therapeutic skills within several domains. With the exception of knowledge related to illness manifestations and concealment of ED behaviors, which was considered important to prevent iatrogenic effects during the stay, all other preferred skills aligned with the psychotherapy literature’s common factors across treatment modalities. One such pan-theoretic domain was motivational enhancement [1, 43–45]. Another involved enhancing knowledge by educating patients regarding the illness, as well as initiating activities to allow opportunities to shift focus during the admission. Looking back, participants seemed to indicate increased desire for staff to take initiative to engage the adolescent despite the family-based focus of treatment, enabling more direct interaction with patients themselves. Additionally, several participants underlined the importance of respect and curiosity, which are acknowledged therapeutic stances. This is in line with the recommended non-judgmental stance characteristic of outpatient FBT [6, 46]. Importantly, at higher levels of care, patients have typically undergone several treatment efforts without experiencing sufficient improvement. Patients may initiate treatment with a lack of trust in the treatment services and presumably, a reinforced view of seeing themselves as a failure [47]. This warrants health care professionals to be especially mindful of how they interact with patients [30, 31, 34]. Interestingly, several of the participants retrospectively reported staff were too lackadaisical or inattentive in recognizing covert ED behaviors, whereas greater awareness could be preventive in the long run [48]. Some patients seem to retrospectively wished behaviors such as water loading or privately excessive exercising in their room had been detected. These reflections underscore that living with AN is not a condition the young person, at least retrospectively, desired. In hindsight, with greater maturity and on average, less afflicted by the ED, findings suggested that the majority called for a greater interest in their own personal views during treatment. The post-treatment interviews seemed to afford the opportunity for participants to caution health care professionals of the potential pitfalls of generalizing too much from theory or previous treatment successes. People are different, and hence, they need individually tailored interventions that accommodate unique qualities and needs.

How exactly increased collaboration with adolescent patients who are ill enough to need hospitalization would look like, is difficult to determine, and represents questions we would like to pursue further. We principally think there is a potential for increasing collaboration with the young patient through all stages of treatment, and that individual variations in severity and impairment along different variables can make arguments for a greater differentiation and a more tailored or personalized treatment during admissions.

### Strengths and limitations

Several strengths and limitations of the study deserve mention. Including all available participants in the analysis (*N* = 37) is considered a strength. Still, potential selection bias cannot be ruled out, as 58 participants were invited to participate. One obvious limitation is the retrospective nature of interviews. The time between hospitalization and the follow-up interview were considerable in length, and thus subject to recall or memory biases. However, a delay between discharge and follow-up may have allowed the participants’ time to reflect sufficiently upon their experiences, and provide greater nuance and self-reflection less affected by events and emotions immediately upon discharge. As the majority of the participants received treatment between discharge and follow-up, we cannot rule out that post-treatment views concerning the family-based admission were influenced by later treatment experiences.

Another limitation is that four of the interviewers were involved in both development and general provision of treatment at the unit, as well as specifically involved in the treatment of some of the participants. This represents a source of bias in the data collection. However, two out of three responsible for analyzing data had no previous work experience at the unit.

The inpatient program and health care setting in Norway enabled the opportunity to provide extended family admissions within a hospital setting, which may limit generalizability to other health care systems. Despite this, we would argue that the study and the findings have proper transferability value [49]. Overall, we would argue that the findings make a contribution to the current literature by improving our knowledge related to patients’ views on important aspects of adolescent AN treatment at higher levels of care. The findings may have implications for treatment development, training and supervision. We believe that the current study can be of relevance for health care professionals and treatment providers offering, or planning to provide, family-based treatment at higher levels of care, both within the ED field and for other psychiatric conditions.

## Conclusion

By investigating former patients’ perspectives pertaining to collaboration and preferred staff behaviors and skills, this study adds to the ongoing work of optimizing the inpatient context for adolescents in need of AN treatment on higher levels of care. Based upon user perspectives from a treatment setting highly influenced by a family therapeutic approach, findings revealed that former inpatients prefer tailored treatment and a collaborative approach. Staff members working within a family-based framework should be equipped with multiple skills and expertise, and clinicians’ knowledge base should not be restricted to family therapy alone. From their unique perspectives as having lived experience and hence, “insider knowledge” with a specific treatment situation, clinicians are reminded of the importance of being mindful on the young persons’ views. Especially, participants raise our awareness of the importance of how we balance between the person and the symptoms, how we balance firmness and flexibility, and overall, how we balance between focusing on the parents and the young person during inpatient family-based treatment for AN.

## Data Availability

The dataset collected and analyzed during the current study are not publicly available as this could compromise participant privacy. The corresponding author can be contacted with questions considering the dataset.

## Abbreviations

AN: Anorexia nervosa
ED: Eating disorder
FBT: Family-based treatment
